# Diet quality among Indigenous and non-Indigenous children and youth in Canada in 2004 and 2015: a repeated cross-sectional design

**DOI:** 10.1017/S1368980021002561

**Published:** 2022-01

**Authors:** Natalie D Riediger, Jeff LaPlante, Adriana Mudryj, Luc Clair

**Affiliations:** 1 Department of Food and Human Nutritional Sciences, Faculty of Agricultural and Food Sciences, University of Manitoba, 209 Human Ecology Building, Winnipeg, MB R3T 2N2, Canada; 2 Department of Community Health Sciences, Max Rady College of Medicine, Rady Faculty of Health Sciences, University of Manitoba, Winnipeg, MB, Canada; 3 National Indigenous Diabetes Association, Winnipeg, MB, Canada; 4 Department of Economics, University of Winnipeg, Winnipeg, MB, Canada; 5 Canadian Centre for Agri-Food Research in Health and Medicine, St. Boniface Albrechtsen Research Centre, Winnipeg, MB, Canada

**Keywords:** Indigenous, First nations, Métis, Diet quality, Healthy Eating Index, Canadian Community Health Survey, Canada

## Abstract

**Objective:**

The objectives were to describe changes in diet quality between off-reserve Indigenous and non-Indigenous children and youth from 2004 to 2015 and examine the association between food security and diet quality.

**Design:**

We utilised a repeated cross-sectional design using both the 2004 and 2015 nutrition-focused Canadian Community Health Surveys, including 24-h dietary recall. Diet quality was estimated according to the Healthy Eating Index (HEI).

**Setting:**

The surveys were conducted off-reserve in Canada’s ten provinces.

**Participants:**

Our analysis included children and youth 2–17 years old (*n* 18 189). Indigenous and non-Indigenous participants were matched, and using a general linear model, we tested time period and (non-)Indigenous identifiers, including their interaction effect, as predictors of HEI.

**Results:**

Both Indigenous and non-Indigenous children and youth had significantly higher HEI scores in 2015 as compared to 2004. There was not a significant (non-)Indigenous and time period interaction effect, indicating the improvements in diet quality in 2015 were similar between both Indigenous and non-Indigenous populations. Improvements in diet quality are largely attributed to reductions in percentage energy from ‘other’ foods, though a disparity between Indigenous and non-Indigenous children and youth persisted in 2015. Overall, food security was lower among the Indigenous population and positively, and independently, associated with diet quality overall, though this relationship differed between boys and girls.

**Conclusions:**

School policies may have contributed to similar improvements in diet quality among Indigenous and non-Indigenous populations. However, an in-depth sex and gender-based analysis of the relationship between food security and diet quality is required.

Current health inequities experienced by Canadian Indigenous populations are a result of colonialism^(1)^. Indigenous People in Canada are comprised of First Nations, Métis and Inuit, as defined in Section 35 of the Canadian Constitution of 1982. Where applicable, we have used the most specific name to refer to the Indigenous group or population. With the release of the Truth and Reconciliation Commission final report in 2015^(1)^, the current federal government has committed to addressing all 94 of the Truth and Reconciliation Commission Calls to Action^(1)^. Number 19 calls to ameliorate disparities in health outcomes, including chronic diseases such as type 2 diabetes^(2–6)^. Importantly, disparities in diabetes vary considerably between First Nations, Métis and Inuit populations, as well as within each population according to region and community^(2–7)^. Furthermore, Call to Action number 20 calls for recognition of, and the need to address, the distinct health needs of Métis, Inuit and off-reserve Indigenous peoples to address jurisdictional disputes for Indigenous people who do not reside on-reserve^(1)^. On-reserve, as outlined by the Indian Act, refers to geographical areas held by the Crown for First Nations and is within federal jurisdiction for healthcare, which is otherwise within provincial jurisdictions. It is important to recognise and address distinct needs for Indigenous people living off-reserve relevant to health, such as food security and diet quality, despite issues pertaining to food being generally outside of healthcare. Diet quality is an important mediating factor for risk of type 2 diabetes, as well as critical for children’s optimal growth and development.

Overall, there have been few recent Canadian studies examining diet quality among off-reserve Indigenous children and youth, and those that have been conducted have reported mixed results. In 2004, Indigenous children, 2–11 years old, living off-reserve were observed to have significantly lower diet quality, or Healthy Eating Index (HEI) scores, as compared to non-Indigenous children, but the difference was no longer significant after adjustment for other factors^(8)^. Results from the 2006 Aboriginal Children’s Survey indicate more than half of Indigenous children 2–5 years old drank juice or soft drinks at least twice per day^(9)^. However, one-third of First Nations children living off-reserve consumed large game animals at least once per month in 2006^(9)^, indicating traditional foods remain an important component of many Indigenous children’s diet, even off-reserve. There is ample evidence supporting the nutritional quality of traditional foods and contributing to cultural and spiritual well-being of Indigenous people^(10)^.

An important predictor of diet quality among children and youth is household food insecurity^(11–13)^, defined here as inadequate access to sufficient, safe and nutritious food due to limited financial means. Food insecurity can also result from, and/or be exacerbated by, limited availability of food, particularly for remote Indigenous communities. Indigenous people living off-reserve are significantly more likely to live in a food insecure household, independent of other socio-economic factors^(14)^. Notably, households with children are more likely to experience food insecurity, particularly lone-parent families^(14)^, and Indigenous children are more likely to live in a lone-parent household^(14)^.

Household income is an important predictor of food security^(14)^. In 2006, the Canadian federal government aimed to provide enhanced income supports to families with children up to 6 years old by introducing the Universal Child Care Benefit^(16)^; this was further enhanced in 2015 to include funds for all children up to 17 years old^(17)^. While these policies were universal and increased absolute financial support to families, they were also less progressive than previous national policy programmes supporting families, such that lower income families did not receive proportionately higher support^(18)^. Therefore, the extent to which income supports are progressive could have implications for equity, including equitable effects on diet quality. Income supplements, such as the more recent Canada Child Benefit (which replaced the Universal Child Care Benefit in 2016), the Ontario Child Benefit and Old Age Security, which are income based, have been shown to be associated with improved food security^(19–21)^, so it follows that income supports directed at families may contribute to improved diet quality, particularly for those experiencing food insecurity.

Since 2004, a number of provincial and school-based policies have also been implemented targeting diet quality and food security among children and youth, including school breakfast and lunch programmes, and policies to reduce access to highly processed foods^(22–24)^. Though not universal, these policies likely contributed to the significantly improved school-hour diet quality scores among Canadian children aged 6–17 years old in 2015 as compared to 2004, as was recently reported^(25)^. During this same time period, an increasing number of Canadian children and youth were also accessing social media with the release of smart phones in 2007^(26,27)^, which has contributed to rapid changes in exposure to health and nutritional information, both credible and non-credible^(28,29)^. An evaluation of diet quality among Canadian children and youth may provide an indication of the extent to which the changing policy and social landscape may have maintained, widened or reduced nutritional inequalities for the off-reserve Indigenous child and youth population. Therefore, the purpose of this study is to describe and test for differences in diet quality between off-reserve Indigenous and non-Indigenous children and youth between 2004 and 2015. A secondary objective is to examine the relationship between food security and diet quality.

## Methods

### Design

We utilised a repeated cross-sectional design using both the 2004 and 2015 Canadian Community Health Survey (CCHS) – nutrition-focused surveys. Both surveys included a general health survey and a single 24-h dietary recall, with a subset completing a second 24-h dietary recall. Response rates were 76·5 and 61·6 % in 2004 (*n* 35 107) and 2015 (*n* 20 487), respectively^(30,31)^. The National Indigenous Diabetes Association (represented by JL) co-led the study including the application for funding; development of and refinement of research questions; informed analysis throughout, including decision-making regarding challenges due to limited sample size; interpretation of the study findings; and ongoing leadership to ensure results are disseminated appropriately. This was achieved through regular face-to-face discussions among the study team and sharing of results.

The 24-h dietary recall follows the United States Department of Agriculture’s 5-step automated multiple pass method, a questionnaire designed to assist in collecting detailed information about the foods and beverages consumed by respondents during the previous 24-h period, from midnight to midnight, to maximise recollection^(31)^. For children aged 2–5 years old, interviews were completed by a parent/guardian, and for children 6–11 years old, interviews were conducted with the assistance of a parent. Children and youth aged 12–17 years old completed the survey and interviews independently.

### Sample

Both the 2004 and 2015 CCHS surveys included a representative sample covering approximately 98 % of the Canadian population (≥1 year old) in the ten provinces. Residents of northern territories (Yukon, Northwest Territories and Nunavut), First Nations living on-reserve, those living in institutions, full-time members of the armed forces, those living in remote areas and Aboriginal settlements were excluded, including Inuit Nunangat. Inuit Nunangat, or the homeland of Canadian Inuit includes Nunavut, but also Inuvialuit, Nunavik and Nunatsiavut^(32)^. Between 2006 and 2016, which were census years, the Aboriginal population increased from 3·8 to 4·9 % of the total Canadian population^(33)^; population growth was attributed to both natural growth and increased self-identification. Métis are most likely to live in an urban area among the three Indigenous groups^(33)^. However, in 2016, an estimated 55·8 % of First Nations people lived off-reserve, with population growth being greater off-reserve compared to on-reserve between 2006 and 2016^(33)^. While the majority of Inuit live in Inuit Nunangat and were not included in the CCHS, the number of Inuit living outside Inuit Nunangat is increasing at a greater rate^(33)^. Therefore, despite the survey exclusions, the results of this study remain valuable to inform policy related to urban Indigenous children and youth. For this study, analyses were limited to participants aged 2–17 years old, which included 12 839 in 2004 (*n* 611 who self-identified as Indigenous) and 5350 in 2015 (*n* 358 who self-identified as Indigenous).

### Measures

Variables included were sex, age, time period, highest level of household education, income adequacy, (non-)Indigenous identifiers and food security status. *Sex* is dichotomised as male and female, or boys and girls. We acknowledge that this variable does not account for the spectrum of sex, nor is it an adequate measure of gender, but it is the only available measure of sex/gender. *Age* was included as a continuous variable. *Time period* is dichotomised as 2004 and 2015, corresponding to the survey years. *Highest level of household education* is categorised as less than secondary school graduation, secondary school graduation and post-secondary education. *Income adequacy*, as defined by Statistics Canada using total household income and number of individuals in the household, was dichotomised as low or mid to high income^(34)^. Importantly, missing data for income adequacy was imputed in both survey years^(31)^. *Household food security status* was operationalised using the Household Food Security Survey Module with respondents categorised as food secure, moderate-food insecure and severe-food insecure^(35)^. The Household Food Security Survey Module contains eighteen questions about the food security situation in the household over the previous 12 months. Questions focus on self-reports of uncertain, insufficient or inadequate food access, availability and utilisation due to limited financial resources and the compromised eating patterns and food consumption that may result. Moderate- and severe-food insecurities were combined to dichotomise responses into *food secure* and *food insecure*. *Indigenous* includes participants self-reporting as sole or mixed Indigenous ancestry (First Nation, American Indian, Métis or Inuit), and all other participants were categorised as non-Indigenous. Indigenous identifiers were further categorised as Métis or First Nations with individuals who identified as both Métis and First Nations categorised as First Nations. Inuit participants could not be reported due to limited sample size and disclosure avoidance but were included within the Indigenous category.

All foods and beverages were analysed using the food composition data from the corresponding Canadian Nutrient File for each survey year^(31)^. The HEI was estimated using an adapted version conforming to the recommendations in 2007 Canada’s Food Guide to Healthy Eating^(8)^. The HEI assesses two aspects of diet quality, adequacy and moderation, including eleven different component scores with a maximum possible score of 100 points^(8)^. HEI is therefore an overall measure of diet quality that is valuable for evaluating nutritional health of the population, assessing trends over time and in comparing different population groups. Usual HEI scores were calculated with the Software for Intake Distribution Estimation programme for the 2004 CCHS^(30)^ and using the National Cancer Institute method for the 2015 CCHS^(36)^. Both methods are used to estimate the distribution of usual intake of a food group or nutrient for a population or subpopulation using the sub-sample who completed two dietary recalls. HEI scores were normally distributed for both Indigenous and non-Indigenous samples in both surveys.

### Statistical analysis

STATA statistical software package version 14 (Stata Corp.) was used and statistical significance set at *α* = 0·05. A sex- and gender-based analysis was also employed such that analyses were stratified by sex. While there were no variables included in the survey to assess gender diversity, gender is considered in the interpretations of the results. All analyses were weighted, according to survey weights provided by Statistics Canada, and variances were estimated using the bootstrapping procedure^(30)^.

First, we determined the proportion of the Indigenous sample in each year who identified as First Nations and Métis. Given known changes in Indigenous self-identification over time, particularly for Métis^(33)^, these proportions are important to the interpretation of the study findings. Second, we described the distributions for the demographic and socio-economic characteristics of the Indigenous and non-Indigenous weighted samples in each survey year, as well as mean HEI. Third, we pooled the survey samples and used a general linear model to test for First Nations and Métis as predictors of HEI. The first model adjusted for age, sex, education, income adequacy, time period and energy intake. We then added food security to the model to determine how the addition of food security impacted the effect size for First Nations and Métis groups on HEI. Additional models were completed using Indigenous identifier (i.e., including First Nations, Metis, and Inuit together) as a predictor of HEI, including an interaction term with time period to determine if the effect of Indigenous identifiers on HEI was dependent on time period. Separate models were also completed for the Indigenous and non-Indigenous samples.

Fourth, using coarsened exact matching, we matched the Indigenous and non-Indigenous samples according to time period, sex, dietary reference intake-based age categories^(30)^, income adequacy and household education^(37)^. Given that Indigenous ancestry is associated with several socio-economic variables that are also associated with diet quality, matching was used to minimise the influence of unobservable confounders and create more comparable groups. Observations for participants in strata without any matches in either the Indigenous or non-Indigenous group were pruned. The remaining matched sample was then reweighted using coarsened exact matching weights^(38)^ and multiplied by the survey weights. We described the matched Indigenous and non-Indigenous samples according to variables matched on to ensure successful matching. Using this matched sample, we fit a general linear model to test for an Indigenous × time period interaction effect on HEI, adjusting for energy intake. Food insecurity was also included as a covariate in a separate model to examine how effect size for the Indigenous population may be influenced by the addition of food security to the model.

Lastly, using the matched samples, we described the mean HEI component scores for Indigenous and non-Indigenous populations in each survey year. To examine more closely what dietary components may be driving changes in overall HEI scores over time, we used independent sample *t* tests to identify any significant differences in mean component scores between the groups within each survey year.

## Results

Off-reserve Indigenous children and youth represent a greater proportion of the Canadian population of children and youth in 2015 as compared to 2004. Furthermore, Métis represent a larger proportion of the Indigenous population in 2015 compared to 2004, though First Nations continue to be the largest of the three Indigenous populations (Table 1). Overall, mean HEI in 2004 was 60·3 (se 0·3) and 62·7 (se 0·4) in 2015. Mean HEI scores among both Indigenous and non-Indigenous children and youth aged 2–17 years old were significantly improved in 2015 compared to 2004 according to weighted, unadjusted analysis (Table 2). Notably, in sex-stratified analysis, only Indigenous girls did not exhibit a significant improvement in diet quality scores in 2015 compared to 2004. Food insecurity remained similarly high among Indigenous children and youth at 28·2 % in 2015, compared to 32·8 % in 2004. In contrast, food insecurity significantly increased in 2015 among the non-Indigenous population to 10·1 %, from 7·6 % in 2004.

**Table 1:** Proportion of samples who self-identify as Indigenous and non-Indigenous, 2–17 years old (% (se))

	Indigenous*					
	Métis		First Nations		Non-Indigenous	
2004 (*n* 12 839)	32·8	3·4	67·2	3·4	97·6	0·2
2015 (*n* 5350)	43·1	4·6	56·9	4·6	95·6	0·4

* Results are reported as a proportion of the Indigenous sample; Inuit participants are included in the Indigenous sample but could not be reported due to small sample size and disclosure avoidance.

**Table 2:** Socio-economic status, household food security and diet quality among Canadian children and youth in the 2004 and 2015 CCHS nutrition surveys (*n* 18 189)

Variables	Indigenous					Non-Indigenous					Matched			
	2004 (*n* 611)		2015 (*n* 358)			2004 (*n* 12 228)			2015 (*n* 4992)		Indigenous (*n* 892)		Non-Indigenous (*n* 15 809)	
	Mean	se	Mean	se	*P*	Mean	se	*P*	Mean	se	Mean	se	Mean	se
Age*	9·7	0·3	9·2	0·4	0·287	9·9	0·03	9·2	0·06	<0·001	9·3	0·3	9·4	0·04
Highest level of household education														
< Secondary school	25·2	3·8	6·7	1·6	<0·001	4·9	0·3	2·4	0·4	<0·001	13·8	1·9	12·5	8·8
Secondary graduation	16·2	3·3	31·7	4·0		11·9	0·4	13·2	0·8		26·3	2·8	23·6	11·2
Post-secondary education	58·6	3·4	61·7	3·9		83·1	0·6	84·4	0·9		59·9	2·8	63·9	20·0
Total household income														
Low	38·4	4·6	14·4	2·8	<0·001	10·5	0·5	6·4	0·6	<0·001	23·0	2·7	25·5	17·2
Middle to high	61·6	4·6	85·6	2·8		89·5	0·5	93·6	0·6		77·0	2·7	74·5	17·2
Food insecurity	32·8	3·7	28·2	3·6	0·05	7·6	0·4	10·1	0·7	0·02	30·3	2·8	16·3	7·5
Energy intake*	2113	85	1870	54	0·012	2589	92	1807	18	<0·001	1965	50	1911	58
HEI*														
Boys and girls	57·7	1·1	61·2	1·2	0·02	61·0	0·2	63·4	0·3	<0·01	59·8	0·8	61·7	0·6
Boys only	*n* 301		*n* 180			*n* 6263		*n* 2516						
	57·1	1·6	61·4	1·3	0·04	60·9	0·3	62·8	0·4	<0·01				
Girls only	*n* 310		*n* 178			*n* 5965		*n* 2476						
	58·2	1·4	61·0	2·0	0·26	61·1	0·3	64·0	0·5	<0·01				

HEI, Healthy Eating Index.

Results presented as percentage (se) with *P*-value corresponding to chi-square test, unless otherwise noted.

* Presented as mean (se) with *P*-value corresponding to *t* test.

A total of 1488 participants were pruned as part of the coarsened exact matching process, resulting in 15 809 and 892 non-Indigenous and Indigenous participants included, respectively. While both populations experienced improvements in diet quality, the difference in diet quality between time periods was not significantly different between Indigenous and non-Indigenous children and youth in either unmatched (Table 3; models 1 and 2) or matched analysis (Table 3; models 5 and 6). Notably, the addition of food security to the model attenuated the effect of Indigenous ancestry, and the interaction effect with time period. Food insecurity was significantly and inversely associated with diet quality, independent of energy intake, (non-)Indigenous ancestry, time period, highest level of household education, income adequacy, sex and age. Matched analysis stratified by sex indicated different associations (Table 3, models 7 and 8), such that the improvement in HEI in 2015 compared to 2004 was only significant among girls, and food insecurity was much more strongly, and negatively, associated with HEI among girls.

**Table 3:** Linear regression coefficient (95 % CI) of Healthy Eating Index for children and youth aged 2–17 years old

	Unmatched†							
	Model 1		Model 2		Model 3 Indigenous only		Model 4 Non-Indigenous only	
	OR	95 % CI	OR	95 % CI	OR	95 % CI	OR	95 % CI
Time period§	2·25***	1·58, 2·92	2·37***	1·71, 3·04	3·14*	0·38, 5·91	2·38***	1·71, 3·05
Indigenous‖	−2·56*	−4·56, −0·57	−1·99	−4·03, 0·04				
Time period × Indigenous	0·94	−1·82, 3·70	0·59	−2·16, 3·33				
Food insecurity¶			−3·43***	−4·52, −2·35	−2·59	−5·61, 0·42	−3·49***	−4·64,-2·33

	Matched‡							
	Model 5		Model 6		Model 7 Girls only		Model 8 Boys only	
Time period§	1·74**	0·63, 2·85	1·78**	0·58, 2·98	2·80***	1·71, 3·89	0·62	−1·50, 2·73
Indigenous‖	−2·96*	−5·43, −0·50	−2·39*	−4·72, −0·06	−1·39	−4·41, 1·62	−3·58*	−6·99, −0·16
Time period × Indigenous	1·41	−2·14, 4·95	1·20	−2·35, 4·74	0·16	−4·87, 5·19	2·61	−2·21, 7·42
Food insecurity¶			−3·18***	−4·50, −1·85	−4·84***	−6·83, −2·84	−1·62	−4·65, 1·41

* *P* < 0·05, **p < 0·01, ***p < 0·001.

† Adjusted for energy intake, sex, age, household education and income adequacy.

‡ Matched according to age, sex, time period, household education and income adequacy; adjusted for energy intake.

§ 2004 as reference group.

‖ Non-Indigenous as reference group.

¶ Food secure as reference group.

First Nations children and youth had significantly lower HEI (−1·93 (95 % CI −3·73, −0·14); *P* < 0·05) compared to non-Indigenous children and youth, independent of time period, energy intake, sex, age, household education and income adequacy. Métis children and youth did not have significantly different HEI scores compared to the non-Indigenous population in the same model (−1·90 (95 % CI −4·36, 0·55)) (data not shown). However, upon further adjustment for food security status, First Nations children and youth no longer had significantly different HEI scores compared to non-Indigenous children and youth (−1·34 (95 % CI −3·11, 0·43)).

Examining component scores of diet quality revealed some significant differences in individual component scores between Indigenous and non-Indigenous children and youth during each time period (Table 4). In 2004, Indigenous children and youth had significantly lower scores on fruit and vegetable intake, dark green or orange vegetables, milk products and percentage energy from ‘other’ foods compared to non-Indigenous children and youth. However, in 2015, the only significantly lower component score between the two groups that remained was for percentage energy from ‘other’ foods, but a significant disparity in Na scores was found in 2015 that was not present in 2004, despite the Na score improving in 2015 compared to 2004 for the Indigenous population. It is important to note that for percentage energy from ‘other’ foods (such as sugar-sweetened beverages, potato chips or candy) and Na, the groups are reverse-scored, meaning that higher intake translates to lower scores. Overall, the greatest absolute improvement in HEI score for the Indigenous population can be attributed to the percentage energy from ‘other’ foods, which the score increased from 10·08 in 2004 to 12·58 in 2015.

**Table 4:** Mean Healthy Eating Index (HEI) component scores (se) among 2–17 year olds using a matched and weighted analysis

Component (maximum score possible)	2004				2015			
	Non-Indigenous		Indigenous		Non-Indigenous		Indigenous	
	Mean	se	Mean	se	Mean	se	Mean	se
Fruit and vegetables (10)	6·11*	0·11	5·48	0·25	5·89	0·30	5·97	0·29
Dark green or orange vegetables (5)	1·11*	0·08	0·74	0·14	1·12	0·09	1·00	0·12
Whole fruits (5)	2·60	0·06	2·26	0·21	2·96	0·15	2·96	0·18
Grain products (5)	4·21	0·02	4·12	0·12	3·90	0·05	3·81	0·12
Whole grain products (5)	1·27	0·09	1·35	0·19	1·00	0·05	0·87	0·13
Milk products (10)	6·78***	0·18	5·67	0·27	6·38	0·30	6·24	0·31
Meat and alternatives (10)	7·50	0·07	7·66	0·33	6·14	0·18	6·07	0·32
Unsaturated fats (10)	8·63	0·07	8·59	0·25	8·28	0·06	8·55	0·20
Saturated fats (10)	6·06***	0·09	6·99	0·23	5·98*	0·16	6·68	0·29
Na (10)	5·23	0·14	4·92	0·33	6·12*	0·09	5·44	0·27
Percentage of energy from “other” foods (20)	11·29*	0·12	10·08	0·55	13·95*	0·16	12·58	0·52

* *P* < 0·05.

*** *P* < 0·001 according to *t* test and as compared to Indigenous population within the same year.

## Discussion

Despite the persistence of food insecurity among Indigenous children and youth, diet quality scores improved in 2015 as compared to 2004. Notably, most of the improvement in HEI scores among both Indigenous and non-Indigenous children and youth can be attributed to improvements in scores for (or more limited intake of) ‘other’ foods, or foods to *limit*, rather than increases in intake for foods Canadians are recommended to *add* such as fruits, vegetables and whole grains. Common ‘other’ foods include candy, chocolate, French fries, alcohol, ice cream and cookies. Dietary changes limiting intake of ‘other’ foods would theoretically be more amenable in the context of food insecurity as opposed to increased intakes of fruits, vegetables and whole grains. Our overall findings are similar to a recent report of diet quality among American children and youth aged 2–19 years old between 1999 and 2016, whereby all racial/ethnic groups experienced similar improvements in diet quality over time^(11)^.

A number of school food policies specifically targeting ‘other’ foods were implemented in many provinces during the early 2000s and 2010s, including banning or improving the nutritional profile of foods in vending machines^(39–45)^, limiting the service of fried foods at school cafeterias^(39–45)^, as well as mandatory physical education, including nutrition education^(46–49)^. However, the implementation of these policies was not universal; therefore, the number of children and youth who may have been impacted is not known. Nevertheless, it appears that efforts to reduce children’s intake of ‘other’ foods have been similarly effective among both Indigenous and non-Indigenous populations, despite persistent food insecurity. However, scores for percentage energy from ‘other’ foods remain significantly lower for Indigenous compared to non-Indigenous children and youth in 2015.

In pooled analysis of both surveys, First Nations and Métis children and youth had similarly lower diet quality as compared to non-Indigenous children and youth, adjusting for other factors; however, with the inclusion of food security to the model, neither population had significantly different diet quality as compared to the non-Indigenous population. Among First Nations children and youth in particular, food security significantly attenuated the association between First Nations and HEI score. These results suggest that food insecurity remains an important determinant of diet quality among Indigenous children and in accounting for differences compared to the non-Indigenous population. Importantly, this relationship differs for Indigenous adults, as we (Riediger *et al.*) have found in, as of yet, unpublished results; Indigenous adults reported significantly lower diet quality compared to non-Indigenous adults, independent of other factors, including food security, and food security was not significantly associated with diet quality among either Indigenous men or women. Taken together, these results suggest that policies to address diet quality among Indigenous children and youth, but particularly First Nations, must address household food insecurity to close gaps in diabetes prevalence for the off-reserve Indigenous populations as called for in the Truth and Reconciliation Commission^(1)^. Importantly, as food insecurity has also been associated with psychological distress among Inuit adolescents^(50)^, alleviating food insecurity among children and youth is likely to have broader improvements for health beyond diet quality.

In sex-stratified analysis, improvements in diet quality in 2015 were limited to girls and without a significant difference between Indigenous and non-Indigenous girls; furthermore, food insecurity was strongly and adversely associated with diet quality among girls. In contrast, for boys, Indigenous boys reported significantly lower diet quality, and food insecurity was not associated with diet quality. Research focused on gender, diet quality and food security among Indigenous children and youth is limited. However, gender differences in diet quality among the Canadian population have been reported such that women and girls reported better diet quality across age groups^(8)^. Relevantly, there is growing research supporting differential gender-based responses to social media with respect to diet^(51,52)^ as well as influences of social media on body image among adolescent girls^(53)^. Perceptions related to body image are also well-known to influence dietary intake among adolescents, including both restrictive eating and other compensatory behaviours to manage body weight^(54–56)^, and high intake of energy dense foods^(57)^. Furthermore, weight stigma is an area requiring greater exploration among Indigenous communities^(58)^, particularly given the emphasis on diabetes prevention and its association with body weight.

Our results presented here pertain to the off-reserve Indigenous population of children and youth and are not representative of First Nations population’s on-reserve or Inuit populations residing in the territories or Inuit Nunangat. Policies to address food security, and subsequently diet quality, for Indigenous children and youth must be developed taking into account the distinct needs of each Indigenous group. However, our results further reinforce the need to ensure food security to improve diet quality among Indigenous children and youth, and subsequently ameliorate diet-related health disparities, such as type 2 diabetes, as called for in the Truth and Reconciliation Commission^(1)^.

Our analysis has multiple limitations, particularly related to data quality. First, the data are not disaggregated by First Nations, Métis and Inuit identifiers other than an initial descriptive analysis and pooled analysis from two surveys at different time periods due to small sample sizes. This is further complicated by the changing composition (First Nations *v*. Métis) in the urban Indigenous population over time and the differing results for First Nations and Métis children and youth for HEI. Furthermore, given the small sample size, we are unable to provide any Inuit-specific data, of which there is little health research available for Inuit living outside Inuit Nunangat. Therefore, pan-Indigenous reporting is a major limitation. Given the small sample size, we were also unable to disaggregate our results into smaller age groups. Another limitation related to the sample is that it may not represent the true off-reserve Indigenous population among the provinces, as Indigenous-specific non-response is not provided by Statistics Canada. Further, the measure of household food security was developed in non-Indigenous contexts and does not fully consider the effects of colonialism, including traditional food practices on food insecurity. The HEI tool used here is not reflective of current dietary guidelines, that is, the new Canada’s Food Guide^(59)^, which does not prescribe specific serving size ranges as the previous guide. However, the HEI is more reflective of the nutritional guidelines in place at the time of data collection. Lastly, the HEI also does not reflect an Indigenous perspective of diet and health. Health Canada is currently in the process of developing an Indigenous-specific food guide, to follow the release of the 2019 general food guide^(61)^.

There are also additional limitations related to the CCHS data quality in both 2004 and 2015, independent of the application to Indigenous populations, which have been outlined by others, including limitations of proxy reporting^(62)^, under-reporting^(63,64)^ and changes in survey methods in 2015 compared to 2004^(64)^, as well as a lower response rate in 2015 (61·6 %) compared to in 2004 (76·5 %), which increase the potential for non-response bias. Lastly, we did not exclude participants with either extremely high or low reported energy intakes. While these results may reflect reporting errors, they may also be realistic given our understanding of food insecurity. However, we did adjust all models for energy intake.

In conclusion, the improvements in diet quality in 2015 compared to 2004 among both Indigenous and non-Indigenous children and youth likely underscore the success of school food policies in reducing intakes of ‘other’ foods and improving diet quality. However, it is likely changes in school food policies, or the income supports for families during this time period, did little to minimise inequities between Indigenous and non-Indigenous populations. In this regard, the improvements in diet quality observed are mostly attributed to foods to limit rather than foods guidelines recommend adding, such as fruits, vegetables and whole grains. This is particularly noteworthy as food security is an important predictor of diet quality and accounts for a significant aspect of the difference in diet quality between First Nations and non-Indigenous children and youth. Further research is needed to examine how food insecurity differentially impacts diet quality of boys and girls. We also recommend future research of dietary interventions or community programmes for both Indigenous and non-Indigenous children and youth utilise sex- and gender-based analysis, as our results suggest boys and girls responded differently to changing policies or social circumstances, and in their dietary responses to household food security.
